# Limited Added Prognostic Value of Choline PET/CT in Treatment-Naïve Hepatocellular Carcinoma

**DOI:** 10.1055/s-0046-1824460

**Published:** 2026-06-10

**Authors:** Irina Vierasu, Hugo Levillain, Martina Pezzullo, Jennifer Dhont, Hugues Azema, Hugo Couvert, Clémentine Marin, Nicola Trotta, Simon Lacroix, Ana-Maria Bucalau, Gontran Verset, Anne Demols, Marianne Paesmans, Desislava Germanova, Valerio Lucidi, Eric Trépo, Thierry Gustot, Christophe Moreno, Jean-Luc Van Laethem, Patrick Flamen

**Affiliations:** 1 Nuclear Medicine Department, Hôpital Universitaire de Bruxelles, Université Libre de Bruxelles, Brussels, Belgium; 2 Medical Physics Department, Hôpital Universitaire de Bruxelles, Université Libre de Bruxelles, Brussels, Belgium; 3 Department of Radiology, Hôpital Erasme, Hôpital Universitaire de Bruxelles, Brussels, Belgium; 4 Department of Gastroenterology, Hepatopancreatology and Digestive Oncology, Hôpital Universitaire de Bruxelles, Université Libre de Bruxelles, Brussels, Belgium; 5 Information Management Unit, Hôpital Universitaire de Bruxelles, Brussels, Belgium; 6 Liver Transplant Unit, Department of Abdominal Surgery, Hôpital Universitaire de Bruxelles, Université Libre de Bruxelles, Brussels, Belgium

**Keywords:** hepatocellular carcinoma, choline PET/CT, [ ^18^ F]FDG PET/CT, MRI, prognostic imaging biomarkers

## Abstract

**Background:**

This retrospective study aimed to assess whether choline PET/CT provides additional independent prognostic value compared with magnetic resonance imaging (MRI) and [ ^18^ F]FDG PET/CT in treatment-naïve patients with hepatocellular carcinoma (HCC). A total of 78 treatment-naïve patients with predominantly single-lesion and early-stage HCC who underwent MRI, [ ^18^ F]FDG PET/CT, and choline PET/CT were retrospectively included.

**Results:**

Higher than median MRI-, choline and [ ^18^ F]FDG PET/CT-derived tumor volumes were associated with poorer overall survival (OS) in univariate analyses. An MRI-derived tumor volume > 17.81 mL was associated with worse OS ( *p* = 0.005; hazard ratio [HR], 3.15; 95% confidence interval [CI], 1.41–7.03), as were choline PET/CT tumor volume > 8.4 mL ( *p* = 0.04; HR, 2.27; 95% CI, 1.03–5.01) and [ ^18^ F]FDG positivity ( *p* = 0.02; HR, 2.74; 95% CI, 1.11–6.81). After multivariate analysis with correction for multiple testing, only MRI-derived tumor volume remained an independent predictor of OS. Although associated with OS in univariate analyses, choline PET-derived metrics did not retain statistical significance after adjustment, suggesting limited incremental prognostic information beyond MRI-derived tumor burden. Liver transplantation and hepatectomy were associated with superior survival compared with locoregional therapies ( *p* = 0.006). No significant correlations were observed between imaging parameters and α-fetoprotein (AFP) levels or ALBI score (all *p* > 0.05). Interaction analyses did not detect any significant modification of the prognostic effect of choline PET imaging biomarkers by AFP levels, ALBI score, or Child–Pugh class. Treatment modality emerged as the strongest prognostic factor in the penalized model using least absolute shrinkage and selection operator regularization, while choline PET-derived parameters did not show independent prognostic value.

**Conclusion:**

Choline PET/CT did not demonstrate additional independent prognostic value beyond MRI or [ ^18^ F]FDG PET/CT in this cohort of treatment-naïve patients with predominantly early-stage and single-lesion HCC.

## Introduction

Liver cancer is the sixth most common malignancy and the third leading cause of cancer-related death worldwide. Hepatocellular carcinoma (HCC) accounts for approximately 90% of primary liver cancers. 1 Magnetic resonance imaging (MRI) is the reference imaging modality for HCC diagnosis and staging, supported by high-quality evidence and international recommendations. 1 2 Tumor size serves as a critical prognostic imaging biomarker alongside features such as arterial phase hyperenhancement, nonperipheral washout, capsule and threshold growth. 1 2 3 A study published in 2024 further demonstrated that MRI-derived tumor size accurately predicts both recurrence-free survival at 2 years and overall survival (OS). 3

According to the most recent European Association for the Study of the Liver (EASL) Clinical Practice Guidelines (December 2024) and the European Society for Medical Oncology (ESMO) guidelines (February 2025), molecular imaging modalities are not recommended for routine tumor staging in HCC. 1 2 Nevertheless, the EASL guidelines recognize [ ^18^ F]FDG PET/CT as a valuable pretransplant tool that can facilitate the selection of candidates for liver resection or transplantation. 1 Similarly, the ESMO guidelines report an association between increased [ ^18^ F]FDG uptake and unfavorable tumor characteristics, including poor differentiation, larger tumor size, elevated serum α-fetoprotein (AFP) levels, and microvascular invasion. While not recommended for routine staging, [ ^18^ F]FDG PET/CT may therefore be useful in selected cases to further characterize findings identified on CT or MRI. 2 However, [ ^18^ F]FDG PET/CT has well-known limitations in HCC. Its sensitivity is reduced in well-differentiated tumors, which predominantly rely on fatty acid metabolism rather than glycolysis. In addition, physiological [ ^18^ F]FDG uptake in normal liver parenchyma may obscure tumor detection, particularly for small lesions. Chronic inflammatory conditions such as cirrhosis may further limit lesion characterization and contribute to false-positive findings.

To overcome these limitations, a potential role for dual-tracer PET/CT using [ ^18^ F]FDG and choline has been proposed. 4 5 6 7 8

Choline is a precursor of phosphatidylcholine, an essential component of cell membrane phospholipids. Increased membrane turnover in HCC cells may therefore result in increased choline uptake, particularly in well-differentiated tumors. 4 9 10 11 Despite encouraging preliminary results, the clinical utility of choline PET/CT in HCC management remains incompletely defined.

Previous retrospective studies including both treatment-naïve and previously treated patients have suggested that dual-tracer PET/CT may lead to Barcelona Clinic Liver Cancer (BCLC) stage migration in a subset of patients, with reported staging upgrades in approximately 11% of cases. 7 Based on these initial reports, choline PET/CT was introduced at our institution as part of the imaging workup for selected HCC patients.

The aim of the present study was to assess whether the combined use of [ ^18^ F]FDG and choline PET/CT provides additional prognostic value beyond standard MRI and [ ^18^ F]FDG PET/CT in a cohort of selected treatment-naïve patients with predominantly early-stage and single-lesion HCC.

## Methods

### Study Population and Imaging Protocol

**Fig. 1:**
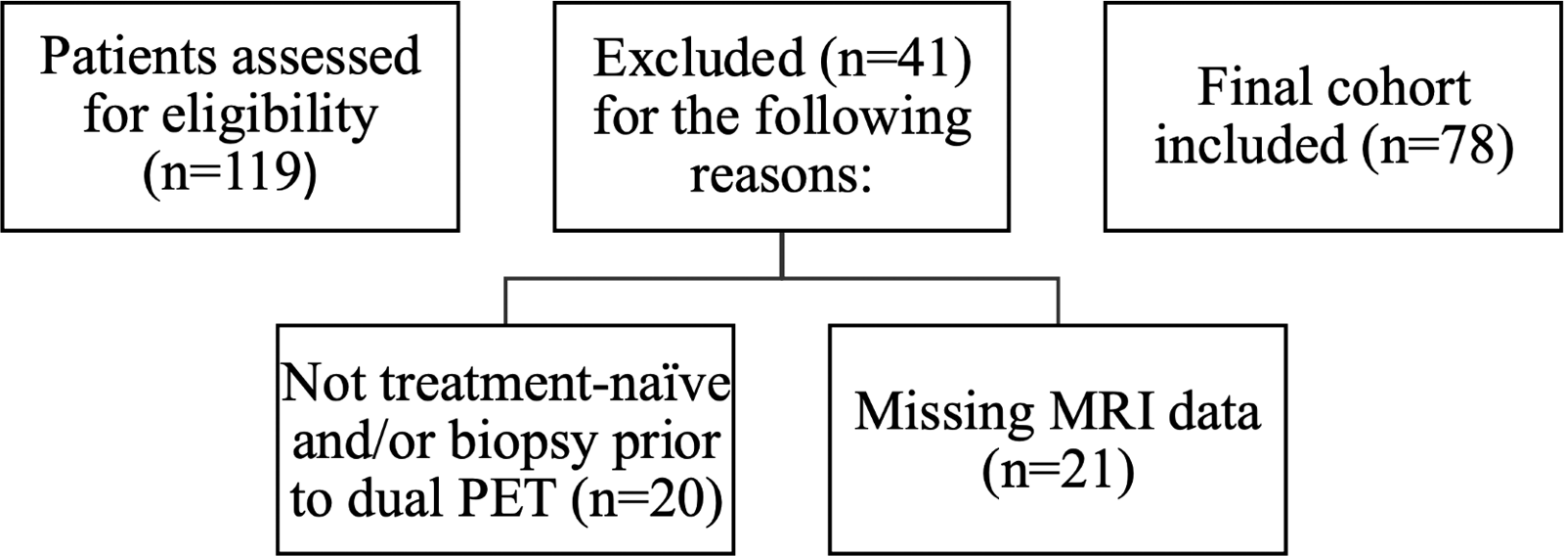
Patient selection flowchart. Patients were retrospectively identified based on the availability of MRI, [ ^18^ F]FDG PET/CT, and choline PET/CT at diagnosis. Reasons for exclusion are detailed.

This retrospective study included patients with MRI features highly suggestive of HCC who were evaluated between 2018 and 2022 in our hospital. Inclusion criteria required patients to be treatment-naïve at the time of imaging, with no prior locoregional or systemic therapy and no tumor biopsy performed before PET/CT acquisition. All patients underwent MRI as well as both [ ^18^ F]FDG and choline PET/CT for initial disease assessment, as illustrated in the patient selection flowchart ( Fig. 1 ). Choline PET/CT was performed in selected patients according to institutional practice, primarily in cases where additional metabolic characterization was considered potentially useful for staging or treatment planning. All consecutive patients fulfilling the eligibility criteria were included.

[ ^18^ F]fluorocholine ([ ^18^ F]FCH) or [ ^11^ C]choline was routinely performed at our institution owing to the availability of an on-site cyclotron. All patients underwent dual tracer PET/CT imaging; no PET/MRI examinations were performed. When [ ^11^ C]choline was available, a 1-day dual-tracer protocol was applied, with [ ^18^ F]FDG injected 40 minutes after completion of the [ ^11^ C]choline acquisition.

PET/CT acquisitions were performed using either a Philips Vereos digital PET/CT system or a Philips Gemini TF-64 PET/CT system (Philips Medical Systems, Cleveland, Ohio, United States). No significant differences were observed in median standardized uptake value (SUV _mean_ or SUV _max_ ) values for choline PET/CT between the digital and analog PET/CT systems, and no significant differences in [ ^18^ F]FDG quantitative parameters were observed between scanners.

Detailed imaging acquisition and reconstruction parameters are provided in Supplementary material (available in the online version only).

MRI, [ ^18^ F]FDG PET/CT, and choline PET/CT were performed within a short time frame at diagnosis to minimize the risk of tumor progression between examinations. The median interval between MRI and choline PET/CT was 8 days (interquartile range [IQR], 4–14), and between MRI and [ ^18^ F]FDG PET/CT was 9 days (IQR, 4–15). The median interval between choline PET/CT and [ ^18^ F]FDG PET/CT was 1 day (IQR, 0–5), with 40% of patients undergoing both PET acquisitions on the same day, 81% within 7 days, and 90% within 14 days. When [ ^11^ C]choline was used, both PET examinations were systematically performed on the same day according to the 1-day dual-tracer protocol.

Clinical data regarding patient age, gender, the presence and etiology of cirrhosis, the biological parameters such as AFP, albumin and bilirubin values, ALBI score, Child–Pugh score, BCLC staging, radiological tumor characteristics, number of lesions, LI-RADS score, oncological tumor board decision and treatments administered were extracted from the electronic health records.

OS was measured in months from the date of diagnosis to the date of death. Patients who were still alive at the time of data extraction were censored at the last follow-up date.

### Image Analysis

**Fig. 2:**
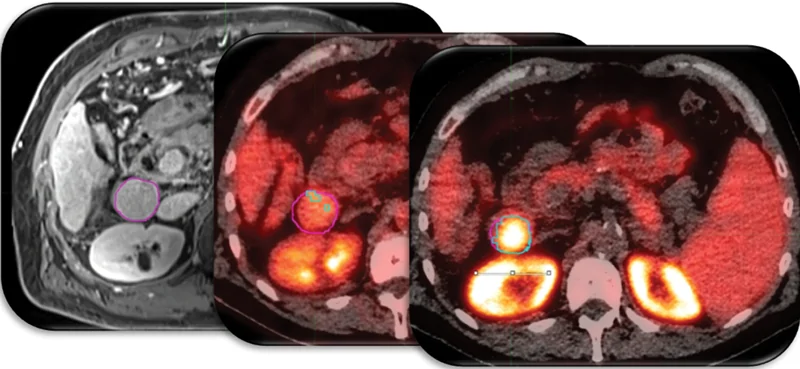
Imaging workflow illustrating tumor volumes defined by MRI, [ ^18^ F]FDG PET, and Choline PET, along with the overlapping regions between modalities in one hepatocellular carcinoma lesion.

MRI and PET/CT images were analyzed using a dedicated MIM 7.3.1-workflow (MIM Software Inc, Cleveland, Ohio, United States), as illustrated in Fig. 2 .

While tumor diameter is the standard-of-care MRI biomarker for HCC, we opted for tumor volume as a surrogate measure as it is also applicable to molecular imaging. Volume offers a more comprehensive assessment of the tumor's three-dimensional extent, especially given the irregular choline volume tumor shape observed in HCC.

The tumor volume of interest (VOI) was manually delineated on MRI and subsequently propagated onto the rigidly registered [ ^18^ F]FDG and choline PET/CT for segmentation. This approach may underestimate metabolically active tumor components extending beyond anatomical boundaries.

To perform segmentation on choline and [ ^18^ F]FDG PET, the workflow permitted the placement of a region of interest (ROI) in the aorta and in the liver. [ ^18^ F]FDG PET segmentation followed the PERCIST criteria, using a reference ROI in the contralateral liver lobe. For choline PET segmentation, no standardized recommendations exist. Thus, we used the mean SUV of the normal liver as a threshold and delineated the ROI in the contralateral liver lobe. We hypothesized that this method is suitable even in cirrhotic livers.

At the end of the workflow, the following parameters were obtained: MRI tumor volume, [ ^18^ F]FDG and choline tumor volumes, mean and maximum SUV (SUV _mean_ and SUV _max_ ) for both choline and [ ^18^ F]FDG. The choline and [ ^18^ F]FDG volumes corresponded to regions within the MRI VOI exhibiting tracer uptake above the defined thresholds, thus representing the active tumor tissue. In cases of multiple lesions, only the largest MRI lesion was used for statistical analyses.

A licensed experienced radiologist validated the VOIs defined on MRI T1 postcontrast acquisitions. The PET segmentations were collaboratively performed by a licensed experienced nuclear medicine physician and a junior nuclear medicine physician, working together on all cases.

### Statistical Analysis

To avoid multicollinearity among tumor volume, the pairwise correlation was analyzed for SUV _max_ and SUV _mean_ obtained through the same imaging modality. For two variables with a correlation coefficient > 0.7, one of the correlated variables was removed from further analyses.

Spearman correlation coefficient was used to assess monotonic associations between MRI, [ ^18^ F]FDG, and choline volumes, whereas Pearson coefficient was used to assess linear association. To assess the individual prognostic value of each remaining imaging parameter and the influence of treatment on OS, univariate Cox proportional hazards regression models were constructed as well as Kaplan–Meier curves.

In both uni- and multi-variate analysis, hazard ratios (HRs) with 95% confidence intervals (CIs) were calculated, and statistical significance was determined using a *p* -value threshold of 0.05, after correcting for multiple-hypothesis testing using the Holm–Bonferroni method. 12

We assessed the correlation between [ ^18^ F]FDG volume and MRI tumor volume within the same tumor for its association with OS. Additionally, the optimal cut-off value for the choline tumor volume as a percentage of the MRI tumor volume was investigated.

To investigate the relationship between AFP, ALBI score and tumor imaging parameters, a Spearman correlation analysis was performed. Interaction analyses were performed to assess whether AFP, ALBI grade, or Child–Pugh class modified the association between choline PET parameters and outcomes.

As a sensitivity analysis, a penalized Cox proportional hazards regression model using least absolute shrinkage and selection operator (LASSO) regularization was performed to assess the stability of prognostic factors and reduce the risk of overfitting, given the relatively limited sample size and number of events. The following characteristics were considered for inclusion in the model: MRI-derived tumor volume, [ ^18^ F]FDG PET-derived tumor volume, choline PET/CT-derived tumor volume, treatment modality, AFP, and ALBI grade. Treatment modality and ALBI grade were entered as categorical variables. Choline PET/CT-derived tumor volume was specifically included as a variable of interest to evaluate its potential incremental prognostic value beyond MRI and [ ^18^ F]FDG PET/CT-derived parameters. The optimal penalty parameter ( *λ* ) was determined using cross-validation, and the *λ*_min_ criterion was selected. Variables with nonzero coefficients at the selected *λ* value were considered retained by the model, indicating a stable contribution to outcome prediction, whereas variables with coefficients shrunk to zero were considered not retained.

## Results

### Patient Characteristics

Between 2018 and 2022, we identified 78 patients (median age: 66.9 years; range: 50–85 years) for study inclusion. The baseline BCLC staging distribution was: BCLC 0, 8 patients; BCLC A, 52 patients; BCLC B, 8 patients; BCLC C, 6 patients; BCLC D, 4 patients. The cohort was predominantly composed of early-stage disease (BCLC 0-A, 60/78 patients, 77%). Most patients had unifocal disease, with 70 of 78 patients (89.7%) presenting a single lesion, whereas 8 of 78 patients (10.3%) had multifocal HCC. Macrovascular invasion was observed in 6 of 78 patients (7.7%), consistent with the proportion of BCLC C patients. Lesions were predominantly classified as LI-RADS 4 or 5, with LI-RADS 5 being the most frequent category among patients with early-stage disease.

The clinical characteristics of the patients are summarized in Table 1 . Of note, only 10 patients were women, and 66 patients had a cirrhotic liver.

**Table 1:** Patients' clinical and biological characteristics

Parameters	Details	*p* -Value
Total patients		78
Gender	Male	68
	Female	10
Age (y)	Median	66.9
	Range	50–85
Cirrhosis	Yes	66
	No	12
Etiology of cirrhosis	Alcohol	28
	Viral Hepatitis	14
	MASLD	7
	Mixed	16
	Hemochromatosis	1
Alpha-fetoprotein (ng/mL)	Median	1,455.7
	Range	3–60,000
Albumin (g/L)	Median	39.3
	Range	0.88–70
Bilirubin (mg/dL)	Median	1.2
	Range	0.23–30
ALBI grade	Grade 1	45
	Grade 2	30
	Grade 3	3
Child–Pugh	A	54 (69.23%)
	B	10 (12.82%)
	C	4 (5.13%)

### Overall Survival and Treatment Modalities

In terms of OS, 51 patients were censored at last follow-up, and 27 died. The median follow-up duration was 24.8 months (range, 1–87). In several subgroups, including patients with lower MRI-derived tumor volume, the Kaplan–Meier survival curves did not cross the 50% survival threshold; therefore, the reported median survival estimates approached the maximum observed follow-up duration of 87 months.

The treatments administered to the patients were heterogeneous: 22 patients underwent liver transplantation (LT), 7 had hepatectomy, 10 received immunotherapy, and 39 received locoregional treatment (LRT) such as ablative therapy (RFA), chemoembolization (TACE), or radioembolization (TARE).

Regarding the influence of treatment on OS, curative approaches like LT and hepatectomy remain the gold standard for eligible patients, providing enhanced survival outcomes. There was a significant statistical difference in OS between the patients who had LT compared with those treated with LRT ( *p* = 0.006).

Choline PET findings did not lead to any modification of treatment allocation in this cohort, as all therapeutic decisions were based on clinical, MRI, and [ ^18^ F]FDG PET/CT findings discussed at the multidisciplinary tumor board.

### Prognostic Value of MRI and Molecular Imaging Parameters

Of the 78 patients included in the study, 74 (95%) demonstrated positive findings on choline PET/CT, characterized by choline uptake in the HCC lesions above the SUV mean of the liver reference ROI. Only 34 patients (43%) showed positive results on [ ^18^ F]FDG PET/CT.

The choice of choline and [ ^18^ F]FDG volumes for correlation with MRI volume was based on the results of pairwise correlation analyses, which demonstrate that volumetric measurements provide more independent and relevant information compared with SUV values, reinforcing their relevance in tumor burden assessment. Choline volume shows a weak correlation with both SUV _max_ ( *r* = 0.22) and SUV _mean_ ( *r* = 0.02). This indicates that choline volume is an independent measure of tumor burden rather than SUV values. [ ^18^ F]FDG volume exhibits a moderate correlation with SUV _max_ ( *r* = 0.37) but remains distinct from SUV _mean_ ( *r* = 0.25). This differentiation supports its inclusion as an independent variable for analysis.

Furthermore, the analysis highlights strong correlations between SUV _max_ and SUV _mean_ in both choline and [ ^18^ F]FDG modalities, suggesting redundancy and multicollinearity; the correlation coefficient was found at 0.94 for choline and 0.97 for [ ^18^ F]FDG.

These findings confirm that SUV _max_ and SUV _mean_ values are highly interdependent, whereas volume metrics provide distinct and complementary insights into tumor characteristics. Therefore, volume-based parameters (choline and [ ^18^ F]FDG) are more relevant for analysis in correlation with MRI volume, as they provide a more comprehensive representation of tumor burden by integrating both metabolic and anatomical characteristics.

**Fig. 3:**
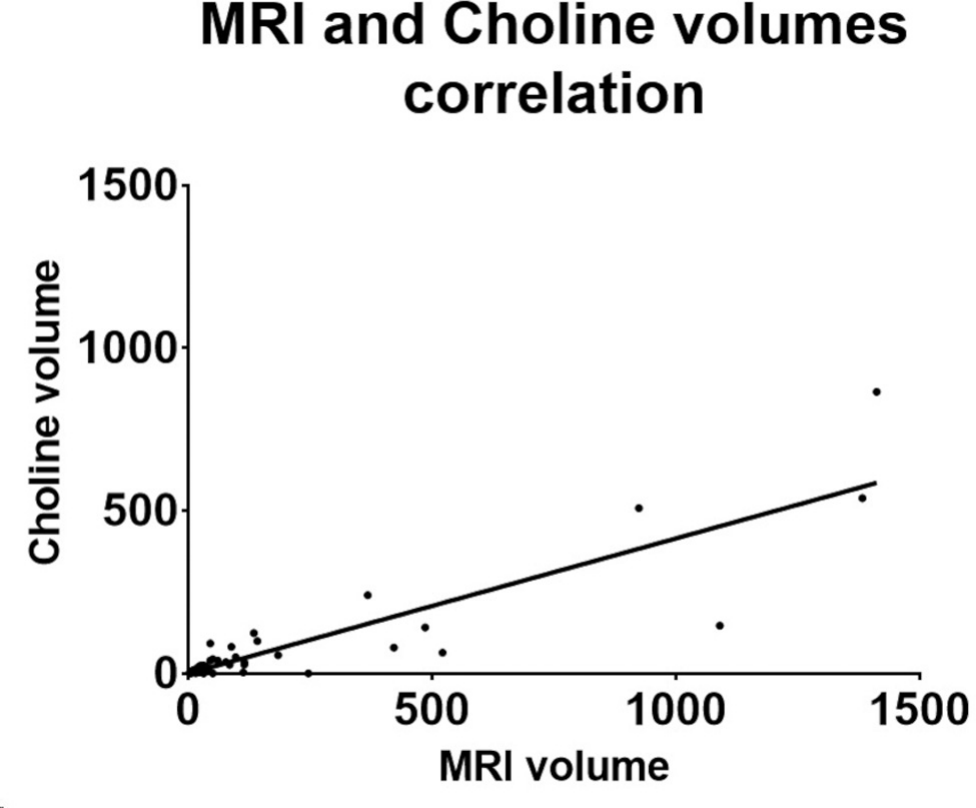
Scatter plot illustrating the association between tumor volumes measured on choline PET/CT and MRI in patients with hepatocellular carcinoma. Pearson correlation analysis ( *r* = 0.8925; *R* ^2^ = 0.7966; *p* < 0.0001).

The results for MRI volume and PET parameters of the lesions are presented in Table 2 . The median MRI, choline, and [ ^18^ F]FDG tumor volumes were 17.81 mL (range: 0.98–1,411), 8.4 mL (range: 0–866), and 0 mL (range: 0–939). A statistically significant association was observed between MRI volume and choline volume ( *R* ^2^ = 0.79; *r* = 0.8925; *p* < 0.0001; Fig. 3 ).

**Table 2:** MRI volume and PET parameters of the lesions

Parameters of the lesions	Mean value ± SD	Number of positive exams (avidity)	Median value + range	Median overall survival (mo) of group below/above dichotomization	Hazard ratio	*p* -Value
MRI volume	114.4 mL ± 278.4	78	17.81 (0.98–1,411)	87/31	3.146 [1.41–7.03]	0.0052
FDG volume	25.02 mL ± 124	34	0 (0–939)	87/26	2.744 [1.11–6.81]	0.02
CHOLINE volume	47.69 mL ± 129.5	74	8.35 (0–866)	87/40	2.270 [1.03–5.01]	0.04
SUV mean choline	9.63 ± 4.32	74	8.94 (4.66–39.5)	40/87	1,173 [0.54–2.54]	0.68
SUV max choline	13.27 ± 6.65	74	12.07 (5.97–54.77)	40/87	1,170 [0.54–2.30]	0.61

The Pearson correlation coefficient ( *r* = 0.57, *p* < 0.0000001) indicates a moderate positive relationship between choline volume and FDG volume.

**Fig. 4:**
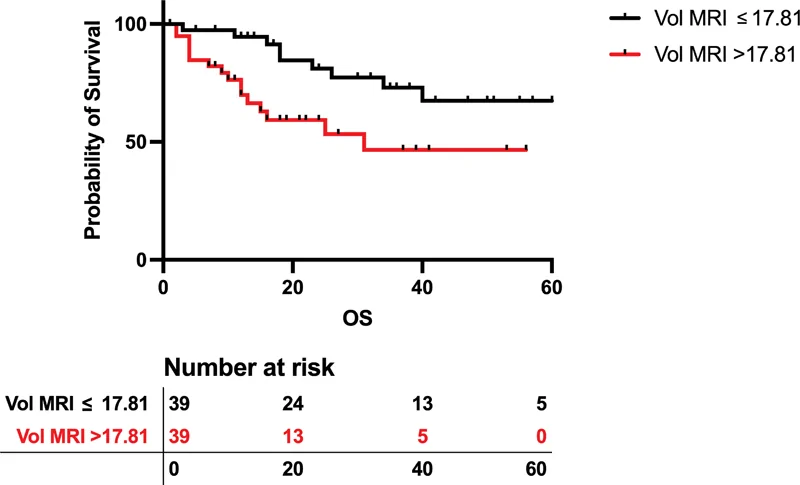
Overall survival (OS) according to MRI-derived tumor volume. Kaplan–Meier analysis illustrating OS stratified according to MRI-derived tumor volume. Patients were dichotomized using the median MRI tumor volume as the cutoff (≤17.81 mL vs. >17.81 mL); *p* = 0.005.

Univariate analyses using Kaplan–Meier curves and the Log-rank (Mantel–Cox) test indicated that higher MRI, choline, and [ ^18^ F]FDG volumes were associated with poorer OS ( Figs. 4 5 6 ). The association between MRI volume, Choline volume, [ ^18^ F]FDG volume, and OS is presented in Table 3 .

**Table 3:** The association between MRI tumor volume, choline tumor volume, [ ^18^ F]FDG tumor volume, and overall survival (OS)

Parameters	Median OS (mo)	*p* -Value
MRI volume ≤ 17.81 mL	87	0.0052
MRI volume > 17.81 mL	31	
FDG volume ≤ 1 mL	87	0.02
FDG volume > 1 mL	26	
Choline volume ≤ 8.4 mL	87	0.04
Choline volume > 8.4 mL	40	

**Fig. 5:**
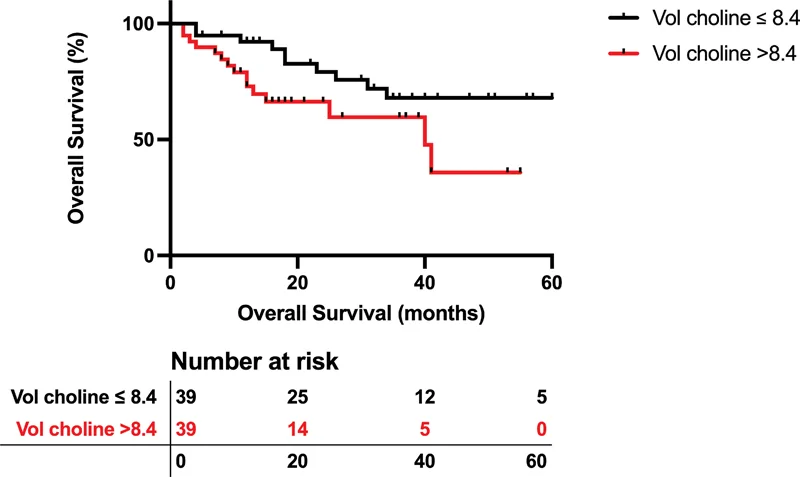
Overall survival (OS) according to choline PET/CT-derived tumor volume. Kaplan–Meier analysis illustrating OS stratified according to choline PET/CT-derived tumor volume. Patients were dichotomized using the median choline PET tumor volume as the cutoff (≤8.4 mL vs. >8.4 mL); *p* = 0.04.

**Fig. 6:**
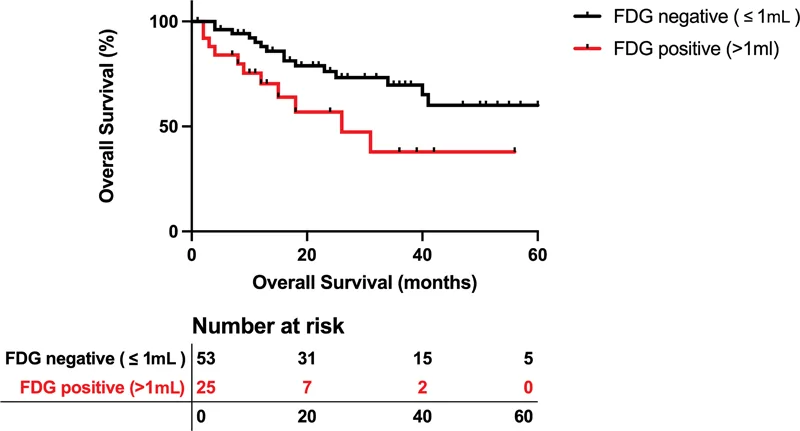
Overall survival (OS) according to [ ^18^ F]FDG PET/CT tumor volume. Kaplan–Meier analysis comparing OS between patients with an [ ^18^ F]FDG PET/CT tumor volume greater than 1 mL and those with a tumor volume of 1 mL or less; *p* = 0.02.

In the group of patients who underwent LT, no significant difference in OS was observed when analyzing either the MRI volume or the choline volume ( *p* = 0.08). When comparing the OS between the [ ^18^ F]FDG ≤ 1 mL and [ ^18^ F]FDG > 1 mL groups, there was not a statistically significant difference for LT patients ( *p* = 0.4).

For [ ^18^ F]FDG PET/CT, a cut-off of 1 mL metabolic tumor volume was applied. This threshold was chosen empirically as the smallest measurable metabolic tumor volume in our segmentation workflow and also emerged as a potential threshold in exploratory analyses of the entire cohort.

**Fig. 7:**
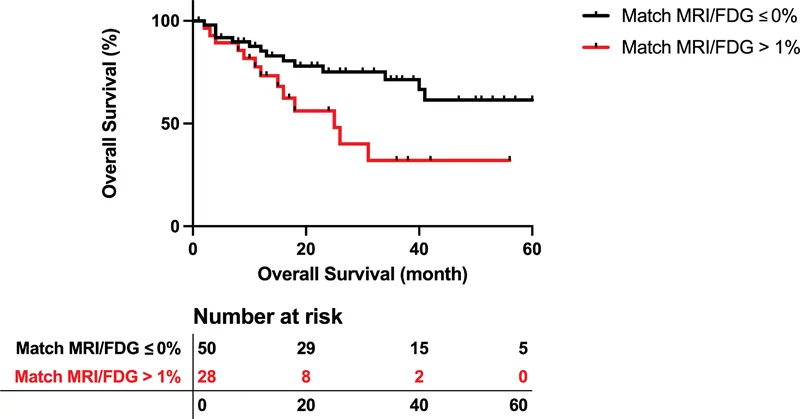
Overall survival analysis according to the concordance between MRI and [ ^18^ F]FDG PET tumor volumes; *p* = 0.01.

Regarding the correlation between [ ^18^ F]FDG volume and MRI volume within the same tumor, a significant association with OS was found ( *p* < 0.01), with an MRI/[ ^18^ F]FDG matching of > 1% (indicating that at least 1% of the tumor shows a significant [ ^18^ F]FDG uptake) linked to poorer OS ( Fig. 7 ).

Exploring the optimal cutoff value, we found that a percentage of choline tumor volume to MRI tumor volume of 55% correlated significantly with OS ( *p* = 0.05). The strongest correlation with OS was observed at a percentage of 60% ( *p* = 0.04).

The median SUV _mean_ for choline was 8.9 (range: 4.66–39.5; SD: 4.32). There was no statistically significant difference in OS between patients with lesions having an SUV mean choline ≥ 8.9 compared with those with SUV mean choline < 8.9.

The mean SUVmean for [ ^18^ F]FDG was 5.03 ± 1.06 (mean ± SD), with a median value of 4.9 (range: 3.53–9.38), and the SUV _max_ for FDG was 6.34 ± 2.28 (mean ± SD).

### Multivariate and Corrected Analyses

Cox multivariate models were developed using dichotomized variables, with median values serving as thresholds. The initial model, titled “MRI + FDG,” revealed a regression coefficient of 1.01, converting to an HR of 2.76, indicating that the occurrence rate of the event was 2.76 times higher in patients with MRI lesion volumes above the median compared with those below it. This coefficient is statistically significant ( *p* = 0.01), suggesting that dichotomized MRI volume is a meaningful predictor of the event risk (death). Conversely, the analysis showed no significant correlation between dichotomized [ ^18^ F]FDG volume and event risk ( *p* = 0.23).

The second model, “MRI + FDG + choline,” included dichotomized choline and [ ^18^ F]FDG volumes, neither of which demonstrated statistically significant predictive value ( *p* > 0.05). However, in this model, dichotomized MRI volume exhibited a trend toward a positive association with event risk ( *p* = 0.09), although this association did not reach statistical significance at the 5% significance level.

To control for multiple hypothesis testing, we applied the Holm–Bonferroni correction to maintain the family-wise error rate at α = 0.05. This method ranks *p* -values from smallest to largest and adjusts the significance threshold in a stepwise manner to reduce the risk of Type I errors. After Holm–Bonferroni correction, MRI tumor volume remained a significant predictor of OS (adjusted *p* = 0.015). [ ^18^ F]FDG PET and choline PET volumes were no longer significant after correction (adjusted *p* > 0.05). The correlation between MRI/FDG matching > 1% and OS remained significant (adjusted *p* = 0.03).

At the *λ*_min_ selected by cross-validation for the LASSO regularization, only treatment-related variables were retained. In contrast, the regression coefficients for MRI-derived tumor volume, [ ^18^ F]FDG PET-derived tumor volume, choline PET/CT-derived tumor volume, AFP, and ALBI grade were penalized to zero and were therefore not retained in the final model.

### Correlation Between Imaging and Clinical Biomarkers

The correlation analysis between imaging-derived tumor parameters and clinical biomarkers revealed weak to negligible relationships across modalities ( Table 4 ).

**Table 4:** Spearman correlation analyses revealed weak to negligible associations between clinical biomarkers (AFP, ALBI) and imaging-derived parameters across MRI and PET modalities

Imaging parameter	AFP ( *r* )	AFP ( *p* -value)	ALBI Score ( *r* )	ALBI Score ( *p* -value)
MRI volume	−0.026	0.821	0.089	0.66
Choline volume	0.063	0.584	0.218	0.05
FDG volume	0.180	0.115	−0.046	0.664
Choline SUV _mean_	0.022	0.855	−0.015	0.899
Choline SUV _max_	0.052	0.660	0.025	0.831
FDG SUV _mean_	0.131	0.459	−0.163	0.357
FDG SUV _max_	0.209	0.235	0.096	0.589

MRI-derived tumor volume showed no meaningful correlation with AFP or ALBI score ( *r* = − 0.026 and *r* = 0.089, respectively).

[ ^18^ F]FDG tumor volume showed a slightly stronger correlation with AFP ( *r* = 0.180), though this did not reach statistical significance ( *p* = 0.115).

Choline SUV _mean_ and SUV _max_ suggested limited relevance in evaluating either tumor burden or liver function. A weak but notable correlation was observed between choline tumor volume and ALBI score ( *r* = 0.218; *p* = 0.05). However, the relationship between choline tumor volume and AFP was negligible ( *r* = 0.063, *p* = 0.584).

The interaction analyses showed that the prognostic effect of choline-derived tumor volume was not modified by AFP levels, ALBI score, or Child–Pugh class.

## Discussion

Our study underscores the limitations of choline PET/CT in providing additional prognostic value compared with MRI and [ ^18^ F]FDG PET/CT for selected treatment-naïve patients with predominantly early-stage and single-lesion HCC.

Despite a strong volumetric correlation between choline PET/CT and MRI, choline PET/CT did not improve prognostic stratification beyond standard MRI or [ ^18^ F]FDG PET/CT. After multiple hypothesis testing correction, MRI tumor volume remained the only statistically significant prognostic imaging biomarker for OS. While choline tumor volume and [ ^18^ F]FDG PET positivity initially showed associations with OS in univariate analysis, these findings lost statistical significance after Holm–Bonferroni correction. The lack of retention of MRI- and FDG-derived variables in the penalized model should not be interpreted as an absence of prognostic relevance, but rather as a reflection of the strong interdependence between tumor burden and treatment allocation. In our study, choline PET/CT-derived parameters did not demonstrate independent prognostic value, including in penalized regression analyses. The interaction analyses showed that the prognostic effect of choline tumor volume was not modified by AFP levels, ALBI score, or Child–Pugh class, further supporting the limited incremental value of choline PET in prognostic modeling.

Among patients who underwent LT, there was no significant difference in OS when comparing MRI and choline volumes.

In our selected patient population, [ ^18^ F]FDG uptake plays a significant role in the assessment of HCC and is strongly correlated with clinical outcomes. We observed a significant statistical correlation between matched [ ^18^ F]FDG and MRI volumes, with an MRI/FDG correlation greater than 1% being linked to inferior OS.

An [ ^18^ F]FDG uptake volume > 1 mL within the MRI-defined lesion could indicate a more aggressive tumor phenotype. While our current data do not permit definitive clinical recommendations, this finding suggests that patients with high [ ^18^ F]FDG uptake could potentially benefit from more intensive treatment approaches, such as combination loco-regional and systemic therapies, or closer posttherapy surveillance including earlier imaging follow-ups. Integrating [ ^18^ F]FDG PET/CT data into multidisciplinary treatment planning could refine prognostic stratification and therapeutic decision-making.

However, in the LT group, while patients with an [ ^18^ F]FDG volume < 1 mL tended to have better OS, the difference compared with those with an [ ^18^ F]FDG volume ≥ 1 mL was not statistically significant. Our findings in the transplanted patient population contradict the data published by Detry et al in 2015, which reported a significant difference in OS between patients with positive and negative [ ^18^ F]FDG PET/CT results. 13 Their study showed that patients with negative FDG PET/CT had significantly better recurrence-free survival ( *p* < 0.001). Moreover, the same study identified the tumor/liver SUV max ratio (RSUV _max_ ), with a cut-off value of 1.15, as a prognostic factor for recurrence and mortality in HCC patients treated with LT.

The observed contradiction between our findings and those reported by Detry et al may be partially explained by the small sample size in our LT subgroup and a potentially more selective patient population. In our study, only three patients had an FDG volume > 1 mL in the LT group, which could have altered the statistical analysis and reduced the power to detect significant differences.

The small number of LT patients with an [ ^18^ F]FDG volume > 1 mL in our study likely reflects a more refined selection process, where patients with high [ ^18^ F]FDG uptake were less frequently considered for transplantation.

Dual-tracer PET/CT has been reported to improve staging and risk stratification in HCC, but our findings did not demonstrate a significant prognostic value of choline PET-derived parameters.

Ghidaglia et al employed machine learning methods to link key histopathological features with dual-tracer PET behavior, illustrating the nuanced biological correlates of choline and [ ^18^ F]FDG uptake. 4 Additionally, Ghidaglia et al conducted a systematic review linking PET uptake behavior to histologic tumor grade. 5 Chalaye et al demonstrated improved tumor staging using choline PET/CT in a clinical subset. 6 Talbot et al highlighted that choline PET uptake correlates with tumor proliferation and membrane turnover. 11 Castilla-Lièvre et al showed that combining choline and [ ^18^ F]FDG PET/CT improves lesion detection in HCC. 8 In 2019, Lu et al provided a comprehensive overview of PET imaging's evolving role in HCC, highlighting its potential integration into radiomics and AI applications. 9

Several factors may explain the discrepancy between our results and some previous studies. Our cohort consisted exclusively of treatment-naïve patients with predominantly early-stage disease. In contrast, previous studies often included more heterogeneous populations, in which tumor aggressiveness may play a more prominent role.

Methodological differences, including acquisition protocols and the timing of dual-tracer imaging, may contribute to variability in PET-derived quantitative parameters across studies. Our analysis focused on the largest lesion only, which may underestimate total tumor burden and intrapatient heterogeneity, both of which are known to influence outcomes.

Also, the use of MRI-derived volume for PET segmentation may underestimate metabolically active tumor components extending beyond anatomical boundaries, particularly in more aggressive lesions. This approach was chosen to allow direct comparison between MRI, [ ^18^ F]FDG PET/CT, and choline PET/CT-derived volumes and to improve reproducibility. [ ^18^ F]FDG uptake, when present, remained a strong indicator of poor prognosis, suggesting that the impact of minor segmentation differences is probably modest. However, our PET segmentation method may introduce selection bias and limit the external validity of our findings.

The use of the liver as a reference tissue presents important limitations, particularly in patients with chronic liver disease or cirrhosis. In such settings, background hepatic uptake may be heterogeneous and influenced by regenerative activity, inflammation, and altered cellular metabolism. While this variability has been extensively documented in [ ^18^ F]FDG PET imaging, similar effects are likely to affect choline-based tracers, although less well characterized. Given that choline uptake reflects membrane phospholipid synthesis and is typically increased in well-differentiated HCC, the surrounding liver parenchyma cannot be considered metabolically inert. This may limit the robustness and reproducibility of liver-based normalization strategies.

We did not perform formal sensitivity analyses using alternative segmentation thresholds for choline PET/CT, which remains a limitation.

The choice of liver-based normalization for choline PET/CT remains a matter of debate, as no standardized threshold for lesion segmentation in HCC has been established. Our choice to use the mean SUV (SUV _mean_ ) of the nontumoral liver as an internal reference was based on both biological rationale and prior literature. In HCC, several studies have relied on tumor-to-liver comparison rather than absolute SUV thresholds. For instance, Yamamoto et al defined HCC lesions as areas of focally increased uptake exceeding that of surrounding noncancerous liver tissue. 10 Recent studies have suggested that choline PET/CT-derived parameters may have prognostic value in specific clinical settings. For example, a prospective study in patients with unresectable HCC treated with immunotherapy reported that higher tumor-to-liver ratios were associated with shorter progression-free survival. However, these findings were obtained in a homogeneous population with advanced disease and uniform treatment, which differs substantially from our predominantly early-stage and treatment-heterogeneous cohort. 14 Semi-quantitative parameters derived from choline PET/CT, including tumor-to-liver ratios, have been used in a study published in 2021 by Donadon et al. 15

The low proportion of patients with multifocal disease in our cohort may have limited the ability to capture the full extent of tumor heterogeneity. In this context, restricting the analysis to the largest lesion is unlikely to have significantly biased the results but may have reduced the potential added value of PET-derived parameters.

Moreover, a thorough review of patient medical records and oncological board decisions revealed that the results of choline PET/CT did not lead to any changes in the management or treatment plan of the patient cohort. No new distant metastases or extrahepatic lesions were identified solely by dual-tracer PET/CT in our cohort. All lesions detected on choline PET/CT were already visualized on MRI, thus limiting the incremental staging value of dual-tracer PET in this population.

This is in alignment with previous studies, including a prospective French multicenter study published in February 2025, which found that dual-tracer PET modified treatment decisions in only 2% of cases (95% CI: 1–5). 16

To summarize the limitations of our study, its retrospective design may have introduced selection bias. Choline PET/CT was performed in selected patients according to institutional practice, when complementary metabolic information was considered clinically relevant for staging or treatment planning. This introduces a significant referral bias and limits external validity. Second, the heterogeneity of treatment modalities may have influenced OS, as treatment allocation is a major determinant of prognosis in HCC and may have overshadowed the impact of imaging-derived biomarkers. Third, residual confounding cannot be excluded, as clinical variables such as AFP level, liver function, and tumor burden are interrelated and may influence survival outcomes. In addition, measurement variability may have been introduced by the absence of standardized segmentation methods for choline PET/CT, particularly in the context of cirrhotic liver parenchyma. Finally, the relatively limited sample size and number of events may have reduced the statistical power to detect modest associations or interaction effects.

Histological confirmation was not systematically available for all patients, as the diagnosis of HCC was often established noninvasively according to EASL and ESMO criteria in patients with cirrhosis and typical MRI findings. For patients undergoing LT or resection, histology was available; however, tumor grade was not integrated into the imaging analysis to maintain consistency across the entire cohort and to avoid selection bias linked to the small histologically confirmed subgroup.

Our data showed that for patients with predominantly early-stage single-lesion HCC who receive standard-of-care MRI and [ ^18^ F]FDG PET/CT, the addition of a choline PET/CT exam is not justified. However, the strong correlation between MRI and choline tumor volumes suggests that choline PET/CT could potentially serve as a substitute for MRI when the latter is unavailable or contraindicated. Further studies incorporating AI-based segmentation techniques and radiomic analyses may refine the clinical role of choline PET/CT in HCC. This possibility warrants further validation and currently does not align with established guidelines.

## Conclusion

These findings are clinically relevant in the context of increasing interest in dual-tracer PET/CT, as they suggest that its routine use in treatment-naïve patients with predominantly early-stage HCC may not be justified when MRI and [ ^18^ F]FDG PET/CT are already available.

In our retrospective study of treatment-naïve patients with predominantly early-stage and unifocal HCC who underwent dual-tracer PET/CT, choline PET/CT-derived tumor volume did not provide additional outcome prognostic value when compared with MRI or [ ^18^ F]FDG PET/CT. Furthermore, no statistically significant correlation was observed between choline SUV max or SUV mean and OS in our patient cohort.

While choline PET/CT may serve as a complementary tool when MRI is unavailable, its routine use in selected early-stage HCC management is not justified based on current evidence.
