# Supplementary material for: Limited Added Prognostic Value of Choline PET/CT in Treatment-Naïve Hepatocellular Carcinoma
Source: World J Nucl Med. 2026 Jun 10;25(3):208–18. doi: 10.1055/s-0046-1824460 (PMC13612106; doi:10.1055/s-0046-1824460)
Supplement: Supplementary file 1 [file 10-1055-s-0046-1824460-s25120013.pdf]

PET/CT Imaging Protocol

$[^{18}F]$FDG, $[^{18}F]$fluorocholine ($[^{18}F]$FCH), and $[^{11}C]$choline were synthesized on site using the hospital cyclotron facilities. All patients underwent PET/CT imaging; no PET/MR examinations were performed.

PET/CT acquisitions were performed on either a Philips Vereos digital PET/CT system or a Philips Gemini TF-64 PET/CT system (Philips Medical Systems, Cleveland, Ohio, United States). Fifty-eight patients were scanned on the Vereos system and twenty on the Gemini TF-64 system during the inclusion period (2018–2022).

$[^{18}F]$FDG PET/CT

$[^{18}F]$FDG PET/CT was performed 60 minutes after intravenous injection of 4 MBq/kg. All patients received intravenous furosemide after tracer injection to reduce urinary tract activity, in accordance with institutional protocols. Whole-body acquisitions were performed from the skull vertex to mid-thigh.

On the Vereos system, acquisitions were performed with a duration of 1 minute 10 seconds per bed position (total acquisition time ~12 minutes), combined with a low-dose, non-contrast CT (30–110 mAs, 110–130 kV). Images were reconstructed using the vendor-provided ordered-subset expectation maximization (OSEM) algorithm (3 iterations, 15 subsets; matrix size 256×256; voxel size $2.67\ mm \times 2.67\ mm \times 4\ mm$).

On the Gemini TF-64 system, acquisitions were performed with 1 minute 30 seconds per bed position (approximately 15 minutes total acquisition time). Low-dose CT was acquired at 120 kV and 50 mA per slice, with a slice thickness of 5 mm. PET images were reconstructed using the default BLOB-based iterative reconstruction algorithm (3 iterations, 33 subsets).

$[^{18}F]$FCH and $[^{11}C]$choline PET/CT

$[^{18}F]$FCH PET/CT was performed after intravenous injection of 4 MBq/kg. Imaging started approximately 10 minutes after injection, corresponding to the steady-state distribution of the tracer in the liver. Whole-body acquisitions (skull vertex to mid-thigh) were performed with 1 minute 30 seconds per bed position on both PET/CT systems, resulting in a total acquisition time of approximately 15 minutes.

When $[^{11}C]$choline imaging was available, a one-day dual-tracer protocol was performed. In these cases, $[^{11}C]$choline PET/CT acquisition started 5 minutes after tracer injection and was performed from head to mid-thigh (8 bed positions), with 1 minute per bed position on the Vereos system and 1 minute 30 seconds per bed position on the Gemini TF-64 system. $[^{18}F]$FDG was injected 40 minutes after completion of the $[^{11}C]$choline acquisition.

Technical Variability

No significant differences were observed in median SUVmean or SUVmax values for choline PET/CT between the digital and analog PET/CT systems. Similarly, no significant differences in $[^{18}F]$FDG quantitative parameters were observed between scanners, suggesting limited technical bias related to scanner type.

 © 2026. The Author(s).
